# Perspective: Nutrition’s Next Chapter – Bioactive Gaps and the Microbiome–Mitochondria Axis

**DOI:** 10.1016/j.advnut.2023.03.016

**Published:** 2023-04-01

**Authors:** Christopher J. Damman

**Affiliations:** Department of Medicine, Division of Gastroenterology, University of Washington, Seattle, WA, United States

**Keywords:** bioactive, microbiome, mitochondria, energy, obesity, malnutrition, double burden

## Abstract

Food has the power to heal. Our bodies transform and are transformed by the elements in food, and the adage that we are what we eat is true. The twentieth century nutrition science focused on decoding the processes and building blocks of this transformation—proteins, fats, carbohydrates, vitamins, and minerals. Twenty-first-century nutrition science is aimed at better understanding the increasingly appreciated bioactive substances within the food matrix that help regulate this transformation—fibers, phytonutrients, bioactive fats, and ferments. Our microbiome and the mitochondria play a key function in orchestrating the role of bioactives in health and are inspiring next-generation nutritional approaches for addressing over- and undernutrition.

**Statement of Significance**

This unique perspective lends a gastroenterological, microbiome, and mitochondrial lens to contextualize advances in nutrition research. It aims to provide a framework for addressing key knowledge and nutritional gaps in our understanding of bioactives that may inform future guidelines, labels, and foods to impact under- and overnutrition.

## All in Perspective – Past Reflections and Future Musings

This last year has seen promising developments in the food-as-medicine movement with the White House’s conference on nutrition announcing the *National Strategy on Hunger, Nutrition, and Health* [1], the NIH’s award of $170 million toward Nutrition for Precision Health Study [2], ramping efforts within The Rockefeller Foundation’s Period Table of Food Initiative [3], a new Food as Medicine research initiative between The Rockefeller Foundation and the American Heart Association [4], and the first-ever dietary bioactive guideline issued for cardiometabolic health [5]. Here, I reflect on next-generation nutrition themes in the context of these developments and experiences having led the Functional Food and Microbiome Initiative at the Bill & Melinda Gates Foundation as well as serving as the chief medical and science officer at an evidence-based functional food company. I aimed to synthesize these experiences that straddle the double burden of malnutrition using the lens of a clinical gastroenterologist with a research focus on the gut microbiome. My hope is to help crystallize a framework for guiding next-generation advances in food-as-medicine.

## Biological Conduits – Framed and Sustained by Nutrition’s Energy

**FIGURE 1:**
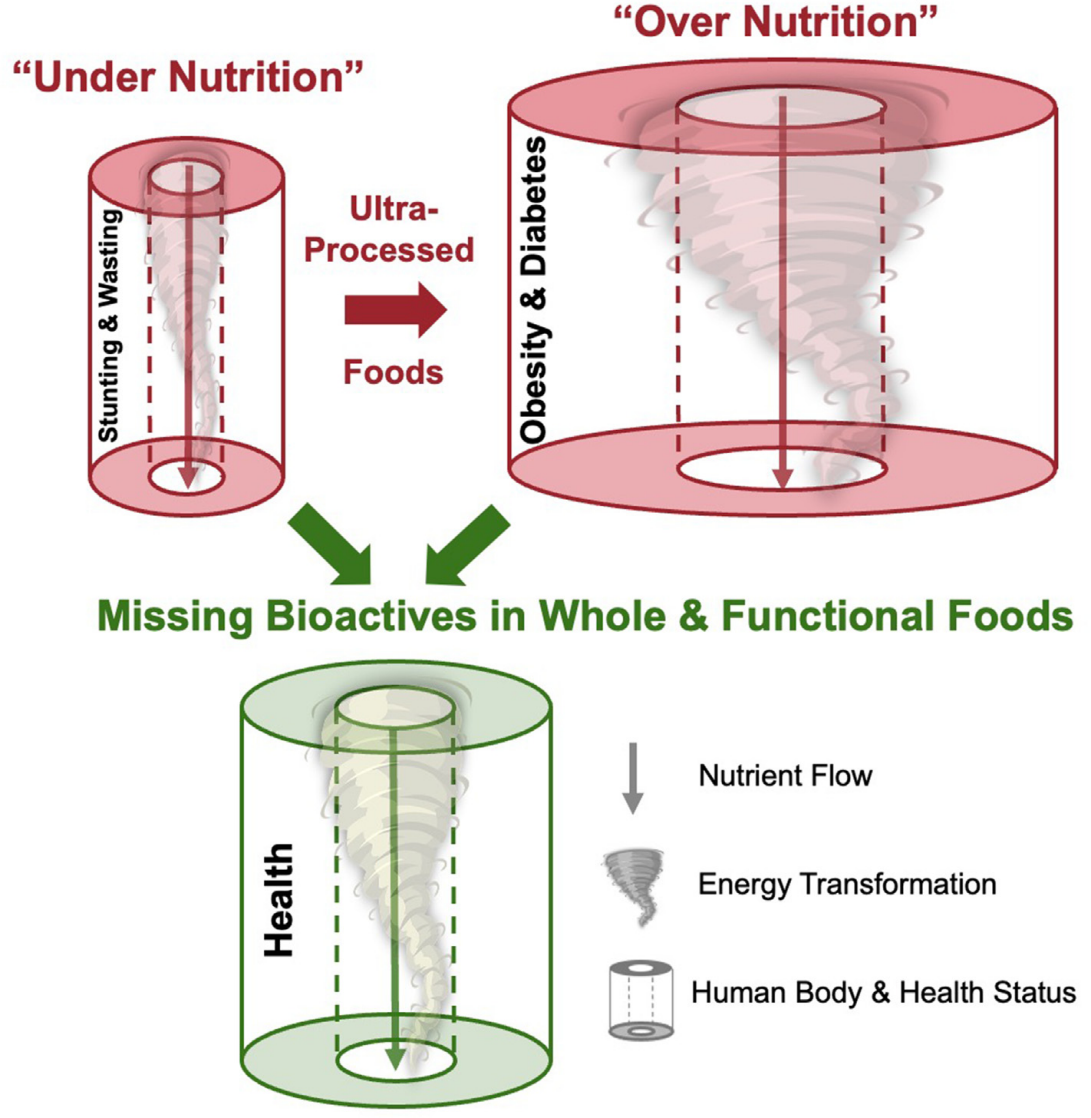
Biological conduits and the double burden of malnutrition. Nutritional energy flows through our bodies, organs, and cells. The depicted vortex is a metaphor for transformation of that energy and the conduits are an abstraction of the emergent individual that is sustained and framed by nutrition. We conventionally think of stunting and wasting as too little nutrition and obesity and diabetes as too much nutrition. This is part of the truth, but healthy nutrition may be more than a Goldilocks phenomenon of macro- and micronutrients. Probably there is also a common denominator to healthy nutrition that is missing on both sides of malnutrition’s double burden. Bioactives in functional and whole foods could be the missing common denominators that help coordinate the transformation of nutritional energy into our healthiest cells and selves.

In our most basic topological body plan, we could be likened to living biological conduits that transform and are transformed by food [6] (Figure 1). This gut-centric simplification throws light on the interplay between nutritional energy and health. The function of the conduits is to process food into energy to grow and sustain vital cells and organs. The result is consistent with the laws of thermodynamics, an overall increase in disorder, but science at the interface of physics and biology has suggested that life is the concentrated pocket of order that emerges from and catalyzes the energy’s transformation [[7], [8], [9]]. Our bodies explained in terms of energy-transforming conduits points to the importance of nutrition to health. Nutrition is in essence the flow of energy through the conduit that frames and sustains it.

## 20th-Century Nutrition – Nutrients and the Double Burden of Malnutrition

The twentieth century nutrition science made significant advancements in decoding the complex components of nutritional processing – understanding how food is broken down into fundamental macro- and micronutrients and how these building blocks can be harnessed to most effectively build the organism that grows, moves, and thinks [10]. The research described the complexities of how food is processed by digestion and metabolic processes such as beta-oxidation, the citric acid cycle, and the electron transport chain to provide ATP to fuel cells.

Modern foods have incorporated these learnings and captured them in nutrition labels that record the specifics of nutritional building blocks—proteins, carbohydrates, fats, vitamins, and minerals. Modern ultraprocessed foods meet the Food and Drug Administration standards for providing certain levels of macro- and micronutrients. Standard ready-to-use therapeutic and supplemental foods in pediatric and maternal malnutrition efforts also capture specified macro- and micronutrient standards [11]. Despite these great advancements, we are learning that some of these foods may contribute to food-associated modern diseases and could be further optimized for healthy growth [12,13]. Indeed, the current secular trend shows stunting and wasting to be on a path to be replaced by obesity and diabetes [14] (Figure 1). Could there be complementary perspectives beyond the current understanding of macro- and micronutrients that are relevant to both sides of malnutrition’s double burden [15,16]?

## 21st-Century Nutrition – Bioactives and Filling the Food Gaps

As the first century of nutrition science was focused on how nutrient energy is broken down into its pieces and reordered to create our cells and organs, the current prevailing questions in nutrition science suggest that the next several decades of research will be focused on learning better how digestive and metabolic processes are *regulated* by the composition and balance of the nutrients in the foods we eat. More specifically, what in food dictates the body’s switch from fighting an infection to growth, fasting to digesting, and hunger to satiety? What regulates metabolic flexibility, insulin sensitivity, and basal metabolic rate? How do the relationships between nutrients and the form they take in food help regulate these processes?

**FIGURE 2:**
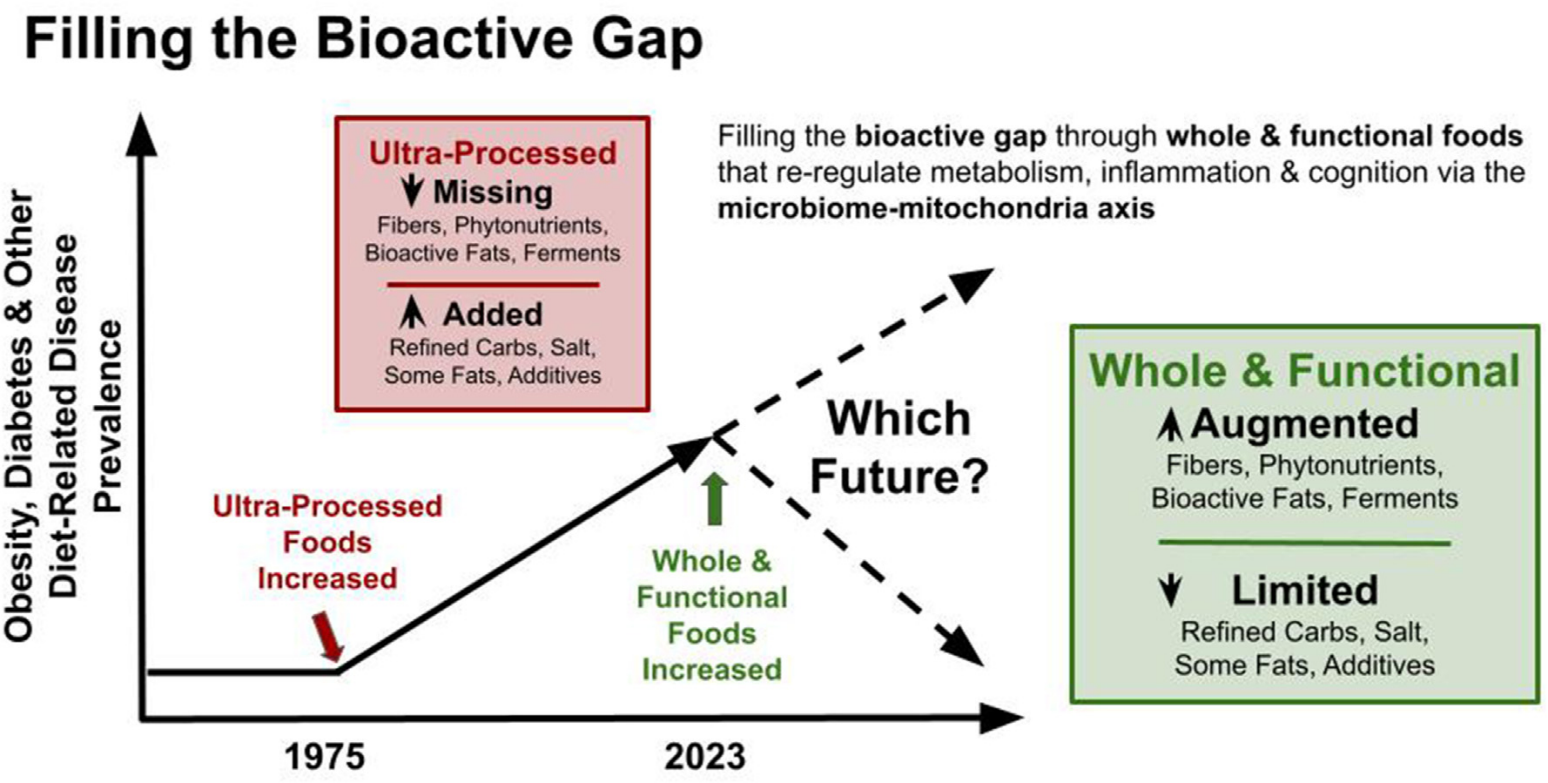
Filling the bioactive gap. Increasing rates of obesity, diabetes, and other diet-associated diseases saw an inflection point in the mid-1970s that correlated with an increase in ultraprocessed foods. One observation is that this correlated with the introduction of added high fructose corn syrup and increased calorie-dense foods. This model posits that in addition to considering what has been added to foods, it is equally important to consider what has been removed, and how the balance of the 2 may ultimately be relevant to promoting health.

These gaps in knowledge may be filled in part by the same gaps that exist in our current modern foods. Due to processing, many bioactive molecules including prebiotic fibers, phytonutrients, bioactive fats, and fermentation products, have been removed or are in less quantities [12,17] (Figure 2). Processing of grains to create white wheat and rice has helped address food insecurity by improving shelf stability and cost of goods, but it has also removed the nutrient- and bioactive-rich bran and germ [17]. In addition, the increase in ultraprocessed foods in both low- and high-income countries has further depleted and disrupted the balance of nutrients, which has been linked to diseases of overnutrition and the double burden of malnutrition [[18], [19], [20]].

Bioactives do not serve the function of conventional building blocks in nutrition, but they are critical for health as regulators of metabolism and other vital life processes. They can be likened to food’s natural “packaging” and biochemical “instructions” to our body for how to most efficiently process nutrients into usable energy. The “packaging” analogy is true in the case of fibers, many of which provide a form to the cell walls of plant cells. The “instructions” are analogous to regulatory signals.

The stunting and wasting research of undernutrition has historically focused on increasing what is missing among macro and micronutrients in impoverished diets—proteins, fats, vitamins, and minerals. The obesity and diabetes research on overnutrition has focused on combinations of limiting what is concentrated in nutrient-dense diets, namely, refined carbohydrates, saturated fats, salt, and additives. Adding to these fundamentally important principles, the latest nutritional research suggests that a common denominator and complementary unlock to metabolic dysfunction in both under- and overnutrition may be bioactives such as fermentable fibers, bioactive fats, phytonutrients, and fermentation products that are largely missing from our diet due to food processing and disruption of the natural food matrix [12,17]. In contrast, bioactive substances are present as abundant components of the diets of exemplar populations (ie, Mediterranean and Okinowan) that live the longest and healthiest lives [21].

To help address the bioactive knowledge gap, the USDA has highlighted the characterization of bioactives such as flavonoids and specific fatty acids as a priority activity for the Nutrient Data Laboratory [22]. Additionally, an expert panel consisting of a collaborative group of nutrition experts with affiliations from the USDA, Academy of Nutrition and Dietetics, and related scientific societies in the field of nutrition (eg, American Society of Nutrition), recently published the first dietary bioactive guideline for cardiometabolic health [5]. This report aligns with the longstanding position of the Academy of Nutrition and Dietetics, which supports the continued research of phytonutrients to promote human health and prevent disease [23,24]. Importantly, the recommendations from recent and classic reports on bioactives can be implemented in the context of current national and international dietary guidelines [[25], [26], [27]].

Also, recently, the Rockefeller Foundation’s Periodic Table of Foods initiative to characterize the metabolomic “dark matter” [28] of foods was launched [3]. Food quality scoring systems that aim to capture elements of the food matrix, including degree of processing, relative ratios of nutrients, and missing bioactives, are being developed [[29], [30], [31]]. Commercial efforts are starting to provide complementary solutions to nutritional guidance such as smart phone applications to personalize and prioritize the whole food selection as well as next-generation products that reincorporate bioactive ingredients into functional foods [[32], [33], [34], [35], [36], [37]].

## The Microbiome–Mitochondria Axis – Maestros of an Energy Symphony

**FIGURE 3:**
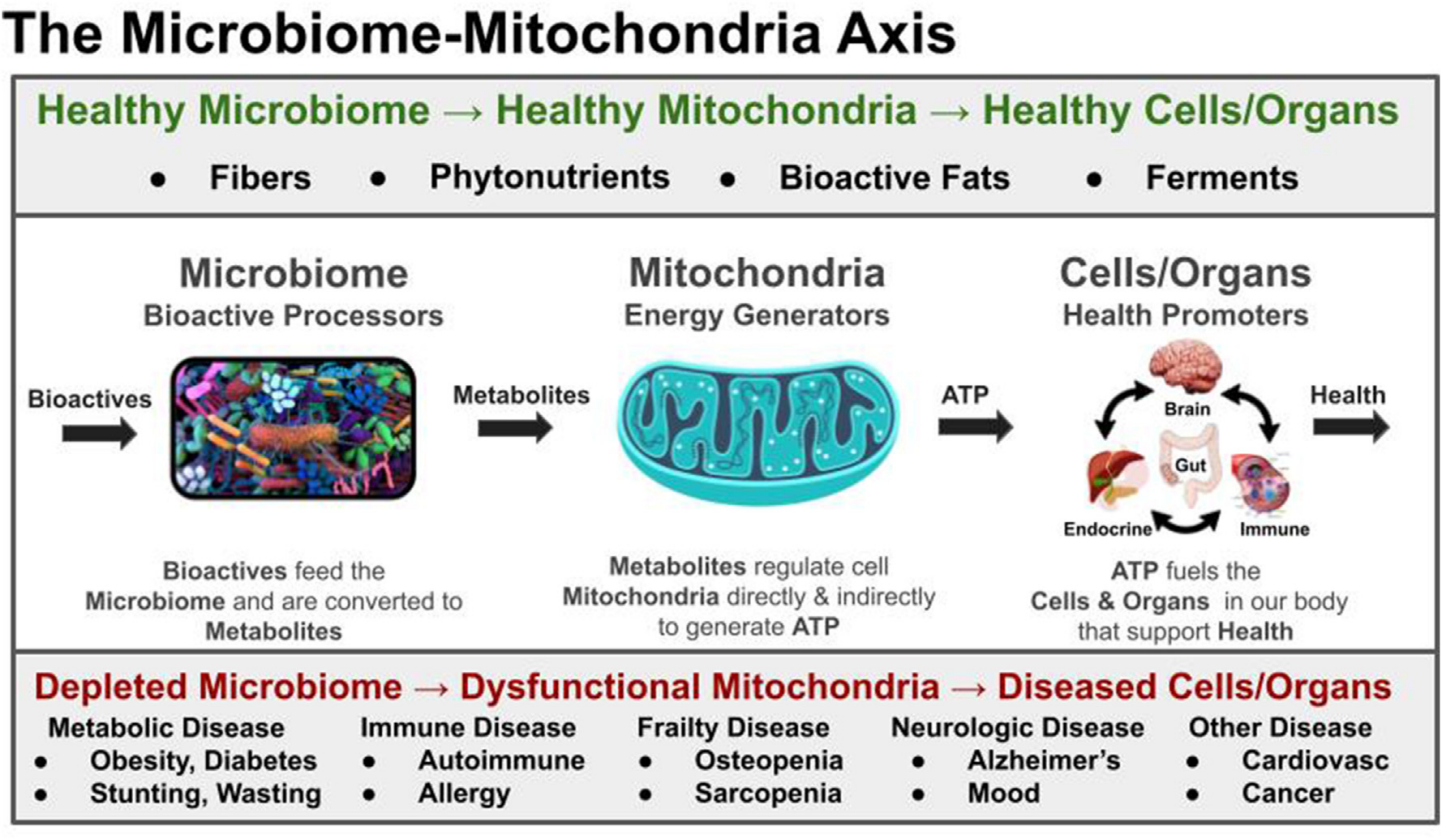
The microbiome–mitochondria axis. Our microbial partners in health are not limited to microbes in our gut. The mitochondria in our cells are believed to have evolutionary roots as alpha-proteobacteria and the interaction between gut microbiome metabolites and cellular mitochondria might be a fundamental mechanism by which our body senses environmental cues, coupling these with ATP generation and expenditure in the body, and helping coordinate energy acquisition and allocation among its diverse organ systems. We are learning that dysfunction in energy processing and signaling by mitochondria is fundamental to many diseases.

Bioactives are not essential in the strict nutritional definition of the word, but they play a fundamental role in regulating healthy growth [38], metabolism [35], appetite [39], immunity [40,41], mood [42], cognition [43], and longevity [44]. They may perform this by directly interfacing with energy processing in our cells [45]. A nascent idea in nutrition science is that the gut microbiome serves as a biological sensor for nutrients and communicates through bioactive signals with their endosymbiotic microbial cousins, the mitochondria [[46], [47], [48]] (Figure 3).

The mitochondria are the cell’s energy powerhouses. They can be likened to the energy maestros of the body, and the bioactive substances, sensed and transformed by their microbiome cousins, can be considered a missing part in their nutritional score. We are learning in the most recent research that the absence of bioactives contributes to mitochondrial and cellular dysfunction in diseases of overnutrition [44,49]. Missing bioactives may also contribute to gut bacterial dysbiosis and breakdown in the body’s defenses against pathogens in diseases of undernutrition [38,[50], [51], [52]].

In overnutrition, food bioactives and mitochondrial dysfunction have been linked to metabolic syndrome, type 2 diabetes, nonalcoholic fatty liver disease, chronic inflammation, hypertension, atherosclerosis, stroke, and Alzheimer’s disease [44,53]. In undernutrition, we are learning the critical role that the microbiome plays in disease pathophysiology. Recently, a microbiome-directed complementary food containing a blend of locally sourced foods (green bananas, chickpeas, soybeans, and peanuts) high in prebiotic fibers, polyphenols, and unsaturated fatty acids was shown to lead to better growth in children with wasting than a standard rice lentil-based complementary food despite the standard complementary food being matched for nutrients and slightly higher in calories [38]. Other studies have linked malnutrition-associated steatosis and leukocyte anergy in malnourished children to mitochondrial dysfunction [50,51].

The microbiome is known to convert fibers, polyphenols, fats, and nucleic acids to short-chain fatty acids [54], modified polyphenols [55], conjugated fatty acids [56], and B vitamins [57]. These metabolites have been shown to regulate mitochondrial genesis and function directly via cellular signaling through epigenetics (HDACi or Sirtuin -> PGC-1alpha), transcription, and translation [54,[58], [59], [60], [61]]. They also communicate indirectly with the distal reaches of the body via paracellular signals such as gut-derived hormones [62,63], cytokines [64], and neurotransmitters [65]. They help connect and coordinate signals in food with the many organ systems of our body. The next-generation in nutrition research is likely to grow our understanding of how these microbial partners in health, working through food bioactives, might play a fundamental role in regulating mitochondrial function, unlocking new approaches to diet-related diseases.

## Nutrition’s Power To Heal – Past Clarity and Future Solutions

There is historical clarity looking back on the development of 20th-century food science that defined nutritional building blocks—the macronutrients, vitamins, and minerals. These perspectives helped solve the root cause of micronutrient deficiency diseases such as beriberi (B1), pellagra (B3), rickets (D), and scurvy (C) [66]. Similarly, we may look back on 21st-century nutrition science and food bioactives as an unlock for understanding and developing solutions for stunting and wasting, obesity and diabetes, and other increasingly recognized food-associated diseases (Figure 3).

In this light, may we continue to embrace research on the interaction of food and its components with our microbial and mitochondrial energy-processing partners in health. May they help guide us on how best to fill some fundamental gaps in our knowledge and nutrition that could inform future guidelines, labels, technologies, and foods.

## Author Contributions

The sole author was responsible for all aspects of this manuscript.

## Author disclosures

CD is on the scientific advisory board at Supergut and BCD Biosciences.
